# The Metabolic and Performance Effects of Caffeine Compared to Coffee during Endurance Exercise

**DOI:** 10.1371/journal.pone.0059561

**Published:** 2013-04-03

**Authors:** Adrian B. Hodgson, Rebecca K. Randell, Asker E. Jeukendrup

**Affiliations:** 1 Human Performance Laboratory, School of Sport and Exercise Science, University Of Birmingham, Birmingham, United Kingdom; 2 Gatorade Sport Science Institute, PepsiCo, Barrington, Illinois, United States of America; University of Bath, United Kingdom

## Abstract

There is consistent evidence supporting the ergogenic effects of caffeine for endurance based exercise. However, whether caffeine ingested through coffee has the same effects is still subject to debate. The primary aim of the study was to investigate the performance enhancing effects of caffeine and coffee using a time trial performance test, while also investigating the metabolic effects of caffeine and coffee. In a single-blind, crossover, randomised counter-balanced study design, eight trained male cyclists/triathletes (Mean±SD: Age 41±7y, Height 1.80±0.04 m, Weight 78.9±4.1 kg, VO_2_ max 58±3 ml•kg^−1^•min^−1^) completed 30 min of steady-state (SS) cycling at approximately 55% VO_2_max followed by a 45 min energy based target time trial (TT). One hour prior to exercise each athlete consumed drinks consisting of caffeine (5 mg CAF/kg BW), instant coffee (5 mg CAF/kg BW), instant decaffeinated coffee or placebo. The set workloads produced similar relative exercise intensities during the SS for all drinks, with no observed difference in carbohydrate or fat oxidation. Performance times during the TT were significantly faster (∼5.0%) for both caffeine and coffee when compared to placebo and decaf (38.35±1.53, 38.27±1.80, 40.23±1.98, 40.31±1.22 min respectively, p<0.05). The significantly faster performance times were similar for both caffeine and coffee. Average power for caffeine and coffee during the TT was significantly greater when compared to placebo and decaf (294±21 W, 291±22 W, 277±14 W, 276±23 W respectively, p<0.05). No significant differences were observed between placebo and decaf during the TT. The present study illustrates that both caffeine (5 mg/kg/BW) and coffee (5 mg/kg/BW) consumed 1 h prior to exercise can improve endurance exercise performance.

## Introduction

Numerous studies to date have shown that caffeine ingested prior to [1]–[7] and during [8] prolonged sub-maximal and high intensity exercise can improve performance. Since the seminal work by Costill and colleagues [9] it is often cited that caffeine induces its ergogenic effects by an increase in fat oxidation through the sympathetic nervous system, and a sequential sparing of muscle glycogen [2]. However, there is very little support for an increase in fat oxidation [10], [11] or an enhancement to the sympathetic nervous system [12] being the principal mechanism of caffeine's ergogenic effect. Since, recent investigations have elucidated that the principal mechanism of caffeine's ergogenic effects is through its ability to act as an adenosine receptor antagonist to induce effects on both central and peripheral nervous system [13] to reduce pain and exertion perception [14], improve motor recruitment [13] and excitation-contraction coupling [15]–[17].

In the literature to date, the ergogenic effects are well documented with the time to exhaustion test at a fixed power output being the predominant performance measure used [1], [2], [9]–[11]. It was questioned whether assessing endurance capacity in this way would have sufficient ecological validity to translate results to real life events [18]. However since then, a number of studies have confirmed the ergogenic effects of caffeine using time trial protocols [3]–[5], [7], [8], which involves completing an energy based target or set distance in as fast as time possible, thus simulating variable intensities that are likely to occur during competitive events. In most of these studies pure (anhydrous) caffeine was ingested through capsules or dissolved in water. Based on this research it is often assumed that ingesting caffeine in a variety of dietary sources, such as coffee, will result in the same ergogenic effect.

Very few studies, however, have shown a positive effect of coffee on exercise performance. Coffee improved performance in some [9], [19]–[21], but not all studies [22]–[24]. This may seem surprising as reports have shown that coffee is the most concentrated dietary source of caffeine as well as being one of the largest sources of caffeine used by athletes prior to competition [25]. Amongst the current studies, only two investigations have actually used coffee rather than decaffeinated coffee plus anhydrous caffeine [21], [22], with only one of these studies showing an ergogenic effect of the coffee [21]. This further identifies the equivocal evidence surrounding the performance effects of coffee. The most cited study is perhaps a study by Graham et al [22], who showed that running time to exhaustion (85% VO_2_ max) was only improved when runners ingested pure caffeine (4.5 mg CAF/kg BW), prior to exercise, but not when they ingested either regular coffee (4.5 mg CAF/kg BW), decaffeinated coffee plus caffeine (4.5 mg CAF/kg BW), decaffeinated coffee and a placebo control. The authors reported that the difference in performance could not be explained by the caffeine or methylxanthine plasma concentrations 1 h following intake or at end of exercise, as no difference was observed between trials that contained caffeine.

Graham et al [22] suggested that other components in coffee known as chlorogenic acids, may have antagonised the physiological responses of caffeine. However, in this study [22] chlorogenic acids in the coffee or in the plasma were not measured. Chlorogenic acids are a group of phenolic compounds that possess a quinic acid ester of hydroxycinnamic acid [26]. The consumption of chlorogenic acids varies significantly in coffee ranging from 20–675 mg per serving [26]. It has previously been shown *in vitro* that chlorogenic acids antagonize adenosine receptor binding of caffeine [27] and cause blunting to heart rate, blood pressure and cause a dose-dependent relaxation of smooth muscle [28]. For this reason, it is unclear what role chlorogenic acids, found in coffee, will have on the physiological and metabolic effects of coffee and caffeine during exercise in humans. Therefore, due to the large variation of chlorogenic acids between coffee beverages and the unclear performance effects of coffee to date, it is yet to be determined if coffee causes differences in the performance and metabolic effects during exercise when compared to caffeine alone.

Therefore the primary aim of the present study was to investigate whether acute intake of coffee (5 mg CAF/kg BW) and anhydrous caffeine (5 mg CAF/kg BW) are ergogenic to cycling performance compared to decaffeinated coffee or placebo beverages when using a validated 45-minute time trial performance test. In addition, completing a steady state exercise bout prior to the time trial performance test is a routine protocol used in our laboratory [18], [29]. For this reason it provided any opportunity to also investigate the effect of acute anhydrous caffeine or coffee intake on substrate oxidation and plasma metabolite responses during 30-min steady state exercise (55% VO_2_ max). The study hypothesis was that despite the previous work by Graham et al [22], 5 mg CAF/kg BW regardless of the form of administration (anhydrous or coffee) would be ergogenic to performance similarly when compared to decaffeinated coffee or placebo, but this effect would not be mediated through changes in fat metabolism.

## Materials and Methods

### Participants

Eight trained cyclists/triathletes (Mean ± SD: Age 41±7y, Height 1.80±0.04 m, Weight 78.9±4.1 kg, 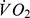
max 58±3 ml•kg^−1^•min^−1^) were recruited from local Birmingham cycling and triathlon clubs. Inclusion criteria included participants who trained 3 or more times per week (>90-min/session), had been training for >2 years, and had a low habitual caffeine intake of ≤300 mg/d (approximately ≤3 cups coffee/d).

### Ethics statement

All participants were fully informed of the experimental trials and all risks and discomforts associated before providing written informed consent to participate in the study. All procedures and protocols were approved by the Life and Environmental Sciences Ethical Review Committee at the University of Birmingham.

### General Study design

The study followed a single blinded, cross over, randomised counter-balanced study design. Maximal oxygen uptake (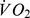
max) and power (Wmax) was assessed during a preliminary trial. Following this each participant completed 4 experimental trials, each separated by 7 days. Each trial consisted of consuming: caffeine (5 mg CAF/kg BW) (CAF), coffee (5 mg CAF/kg BW) (COF), decaffeinated coffee (DECAF) or placebo (PLA) in the overnight fasted state (8 hrs) 1 h before completing 30-min steady state cycle exercise bout (SS) (55% 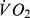 max). Following this each participant was instructed to complete a time trial lasting approximately 45-min.

### Preliminary Trial

Before the experimental trial, participants visited the Human Performance Laboratory at the University of Birmingham on two separate occasions separated by 7 days. During the first visit participants completed an incremental exercise test on an electronically braked cycle ergometer (Lode Excalibur Sport, Groningen, Netherlands) to volitional exhaustion (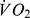
max test). Prior to beginning the test participants firstly had weight (OHaus, Champ II scales, USA) and height (Seca stadiometer, UK) recorded. Participants mounted the cycle ergometer, which was followed by a 5-min warm up at 75 W, participants then started the test at 95 W for 3-min. The resistance was increased every 3-min, in incremental steps of 35 W, until they reached voluntary exhaustion. Wmax was calculated using the following equation:
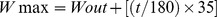



Where W_out_ is the power output of the last stage completed during the test, and t is the time spent, in seconds, in the final stage. Throughout the test respiratory gas measurements (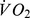 and 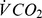) were collected continuously using an Online Gas Analyser (Oxycon Pro, Jaeger). 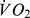 was considered maximal if 2 out of the 4 following criteria were met: 1) if 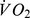 levelled off even when workload increased 2) a respiratory exchange ratio (RER) of >1.05 3) a heart rate within 10 beats/min of age predicted maximal heart rate 4) a cadence of 50 rpm could not be maintained. Heart rate (HR) was recorded during each stage of the test using a HR monitor (Polar, Warwick, UK). Wmax was used to determine the work load for the steady state exercise bouts throughout all subsequent experimental trials (50% W_max_). Wmax was also used to calculate the total amount of work to be completed during the 45-min time trial and the linear factor, both calculated according to the formula derived by Jeukendrup et al [18]. The bike position was recorded following the test to be replicated in all other trials.

Approximately 7 days later participants reported back to the lab, for the second preliminary trial, between 0600 and 0800 having undergone an 8 hr fast. The purpose of the trial was to familiarise each subject to the experimental trial and time trial protocol. All participants completed 30-min SS at 50% *W*_max_ (55% 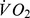
max). Expired breath samples were collected every 10-min for measures of
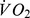
_,_ 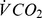
_,_ and RER (Oxycon Pro, Jaeger). Immediately following all participants completed a time trial lasting ∼45 min. The data collected during the familiarisation trial was not used for any of the final analysis.

### Experimental Trial

All participants reported to the Human Performance Lab between 0600 and 0800 having completed an 8 h overnight fast. On arrival weight was recorded (Seca Alpha, Hamburg, Germany) and a flexible 20-gauge Teflon catheter (Venflon; Becton Dickinson, Plymouth, United Kingdom) was inserted into an antecubital vein. A 3-way stopcock (Connecta; Becton Dickinson) was attached to the catheter to allow for repeated blood sampling during the experimental period. An initial 15 ml fasting blood sample was collected (EDTA-containing tubes, BD vacutainers). Following this all participants consumed one of the treatment beverages and rested for 1 h, with further samples taken at 30 min and 60 min (10 ml EDTA). After the rest period, participants then mounted the cycle ergometer, in an identical bike position as recorded during the preliminary trial, and began a 30-min SS at 50% *W*_max_ (55% 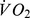
max). Blood samples (15 ml) and 5-min respiratory breath samples, VO_2_, VCO_2_ and RER, (Oxycon Pro, Jaeger) were collected every 10-min during the exercise. The catheter was kept patent during both the rest and exercise period by flushing it with 5 mL isotonic saline (0.9% w/v; B Braun) after every blood sample. In addition, heart rate (HR) was recorded (Polar RS800CX) every 15 min at rest and every 10 min during SS. Ratings of Perceived Exertion (RPE) scale were recorded every 10 min during SS using the 6–20 Borg scale [30]. Upon completion of the SS, the subject was instructed to stop exercising for ∼1 min, and the cycle ergometer was set in the linear mode. The participants were instructed to complete an energy-based target amount of work at 70% W_max_ in the quickest time possible. The total amount of work (650±37 KJ) was calculated for the 45 min time trial. A linear factor, 70% W_max_ divided by (90 rpm)^2^ was entered into the cycle ergometer. The time trial protocol employed has previously been validated and has been shown to be highly reliable [18]. Participants received a countdown prior to starting the time trial and received no verbal or visual feedback regarding performance time or physiological measures throughout the test. No additional measures, blood or respiratory, were taken through the test. Participants received no feedback about their performance until they had completed all 4 experimental trials. Following the completion of the TT, each participant completed a questionnaire to guess the test beverage consumed prior to the commencement of the trial, as well as report any GI distress experienced during the trial.

### Treatment Beverages

During each visit to the lab, participants ingested one of four treatment beverages. This included caffeine (5 mg CAF/kg BW), regular coffee (5 mg CAF/kg BW), decaffeinated coffee and placebo. Therefore decaffeinated coffee and placebo acted as controls to both of the caffeinated trials. Caffeine (Anhydrous caffeine, 99.8% pure, Blackburn Distributions Ltd, Nelson, United Kingdom) was weighed (394.4±7.0 mg) prior to the trial, and was immediately dissolved and vortexed for 15 min in 600 ml of water prior to consumption and served in an opaque sports drinks bottle. Coffee was prepared using instant coffee (Nescafe Original). In order to select the correct weight of coffee to equal 5 mg CAF/kg BW, Nescafe states that Nescafe Original instant coffee provides 3.4 g caffeine/100 g of instant coffee. This information was confirmed using a HPLC method (see below), and based on the analysis it was calculated that 0.15 g coffee/kg/BW equalled 5 mg CAF/kg BW. Therefore prior to each trial, coffee was weighed (11.8±1.0 g) and dissolved in 600 ml hot water (94±2°C) and served in a mug.

DECAF (Nescafe Original Decaffeinated coffee, <97% caffeine free) was prepared in an identical fashion, with the same amount of decaffeinated coffee as the COF beverage. Using a HPLC method, decaffeinated coffee provided minimal caffeine throughout each of the prepared beverages (0.17 mg CAF/kg BW or mean intake of 13.41±0.70 mg). In order to blind the participants from the taste of the caffeine trial, the placebo trial consisted of 8 mg of Quinine sulphate (Sigma, UK). Quinine sulphate is a food ingredient found in tonic water to give a bitter taste. The quinine sulphate was dissolved in 600 ml of water, vortexed for 15 min, and served in an opaque sports drinks bottle. The exact dose of quinine sulphate was preliminary tested in our lab. A dose of 8 mg was sufficient to prevent the blinded researchers from distinguishing between the placebo and caffeine trial.

The coffee and decaffeinated coffee samples were further analysed externally for chlorogenic acids (5-CQA) (Eurofins Scientific, Italy). Based on the analysis, total 5-CQA was 33.91 mg/g and 28.29 mg/g for COF and DECAF respectively. This was then used to calculate the average concentration of total 5-CQA and related isomers for each participants drinks, based on the weight of instant COF and DECAF. The average chlorogenic acid intake in COF and DECAF are presented in Table 1. All of the beverages were prepared in 600 ml of water. This was firstly to avoid any difference in uptake and bioavailability of each of the ingested ingredients, as well as replicating the format of coffee consumption from everyday life. Secondly 600 ml of coffee dissolved in water was not only considered tolerable, based on taste testing by researchers within our laboratory, but also matched similar volumes as used by Graham et al [22]. Once participants received each of the beverages at the beginning of the trial, they had 15 min to consume the entire 600 ml.

**Table 1:** Mean caffeine and chlorogenic acid (Total 5-QCA) concentration in each treatment beverage serving.

Treatment beverage	Serving Volume (ml)	Caffeine content (mg/serving)	Total 5-CQA (mg/serving)	% of Total 5-CQA								
				CQA	5-CQA	4-CQA	5-FQA	4-FQA	3,5-diCQA	3,4-diCQA	4,5-diCQA	4,5-CFQA
CAF	600	394.4±7.0	-	-	-	-	-	-	-	-	-	-
COF	600	394.4±7.0	393.3±7.3	21%	32%	22%	8%	7%	3%	2%	3%	2%
DECAF	600	13.4±0.2	328.1±6.1	23%	33%	24%	7%	7%	3%	1%	2%	1%
PLA	600	-	-	-	-	-	-	-	-	-	-	-

Abbreviations: CQA Caffeoylquinic acid, 5-CQA 5-O-Caffeoylquinic acid, 4-CQA 4-O-Caffeoylquinic acid, 5-FQA 5-O-Feruloylquinic acid, 4-FQA 4-O-Feruloylquinic acid, 3,5-diCQA 3,5-O-Dicaffeoylquinic acid, 3,4-diCQA 3,4-O-Dicaffeoylquinic acid, 4,5-diCQA 4,5-O-Dicaffeoylquinic acid, 4,5-CFQA 4,5-O-Dicaffeoylquinic acid, ml millilitres, mg milligrams, CAF Caffeine, COF Coffee, DECAF Decaffeinated Coffee, PLA Placebo.

### Diet and Exercise Control

Participants were instructed to record their food intake the day prior to the preliminary familiarisation trial. Participants had to replicate this diet in the 24 h prior to each experimental trial, as well as refraining from any exercise, consume no alcohol and withdraw from any caffeinated products.

### Calculations

Substrate metabolism was measured during the SS. From the respiratory output measurements of 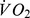
and 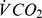 (L/min), carbohydrate [1] (CHO) and fat oxidation [2] was calculated every 10 min during the SS. In order to calculate CHO and fat oxidation stoichiometric equations [31] were used, which assume that each of the participants were exercising at a steady state and that protein oxidation was negligible.
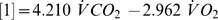


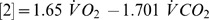



### Blood Analysis

Following collection, all tubes were placed in ice until the end of the experimental trial. Following this each tube was centrifuged at 3500 rpm for 15 min at 4°C. Aliquots of plasma were immediately frozen in liquid nitrogen and stored at −80°C for later analysis. Each blood sample taken throughout each experimental trial were analysed for plasma glucose (Glucose Oxidase; Instrumentation Laboratories, England), fatty acids (FA) [NEFA-C; Randox, England], glycerol (Glycerol; Randox, England) and lactate [Lactate, Randox, England] using an ILAB 650 (Instrumentation Laboratory, Cheshire, United Kingdom).

### Plasma Caffeine and Chlorogenic Acid analysis

Plasma caffeine were analysed externally (City Hospital, Dudley, Birmingham) using a reversed-HPLC-UV method. The sample preparation included: 200 µL of plasma were added to 100 µL internal standard (Proxyphylline, Sigma, United Kingdom) before mixing, heating and adding 500 µL of acetic acid. The supernatant was the injected (5 µL) onto a Phenomenex Prodigy 150×4.60 mm 5 µ Octadecyl Silane (ODS) column using an auto sampler and detected at a UV of 273 nm. Caffeine concentrations were quantified using one point calibration from a calibrator that had previously been internally validated against a 9 point calibration curve (City Hospital, Dudley, Birmingham). With each batch two QC (High and Low) were run, with reference to the internal standard to account for any loses.

The caffeine content of coffee and decaffeinated coffee were confirmed at the School of Sport and Exercise Sciences. In brief, coffee and decaf coffee samples were prepared (identical to preparation described above) and cooled before 5 µL of each sample were injected onto a Phenomenex Luna 10 µ C_18_ (2) column using an auto sampler (WPS 3000, Dionex, United Kingdom). The mobile phase consisted of 0.1 M acetic acid in water and 0.1 M acetic acid in acetonitrile. Caffeine concentrations were quantified using a 10-point caffeine calibration curve in water.

Coffee and Decaf coffee were analysed for chlorogenic acids. The analysis was conducted externally (Eurofins Scientific, Italy) using a reverse HPLC methodology at 325 nm (Water Symmetry C18, 250×4.6 mm, 5 µm) with external 5-QCA standards for quantification on a 3 point calibration (10–250 mg/kg). The mobile phase consisted of aqueous 0.5% formic acid and acetonitrile.

### Statistical Analysis

Data analysis was performed using SPSS for WINDOWS software (version 17; SPSS Inc, Chicago, IL). Data are expressed as means ± SEMs, unless otherwise stated. A repeated measure ANOVA was used to assess differences in respiratory, substrate metabolism, plasma metabolite and caffeine concentration as well as time trial performance measurements during each trial. In order to detect differences across time and between treatments a Fisher protected least significant differences post hoc test was used Significance was set at P<0.05.

## Results

### Steady State Exercise

#### Whole body respiratory measures, HR and RPE

The selected workload of 50% Wmax during the SS (171±7 W) resulted in similar oxygen uptake (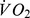) (2590±79, 2595±89, 2465±79, 2522±71 mL/min for CAF, COF, DECAF and PLA respectively P = 0.278). As a result the relative exercise intensity during the SS was similar throughout each trial (58±2%, 58±2%, 55±1% and 55±1% for CAF, COF, DECAF and PLA respectively P = 0.337). Energy expended during SS was also shown to be similar (1607±49 KJ, 1611±54 KJ, 1531±50 KJ, 1565±43) for CAF, COF, DECAF and PLA respectively P = 0.248). In addition no significant difference was observed in average HR during exercise (119±4, 119±4, 119±4, 120±5 bpm for CAF, COF, DECAF and PLA respectively P = 0.281) or RPE values (10±0, 10±0, 11±0 and 11±0 for CAF, COF, DECAF and PLA respectively P = 0.091) during SS between trials.

#### Carbohydrate and Fat oxidation

**Figure 1:**
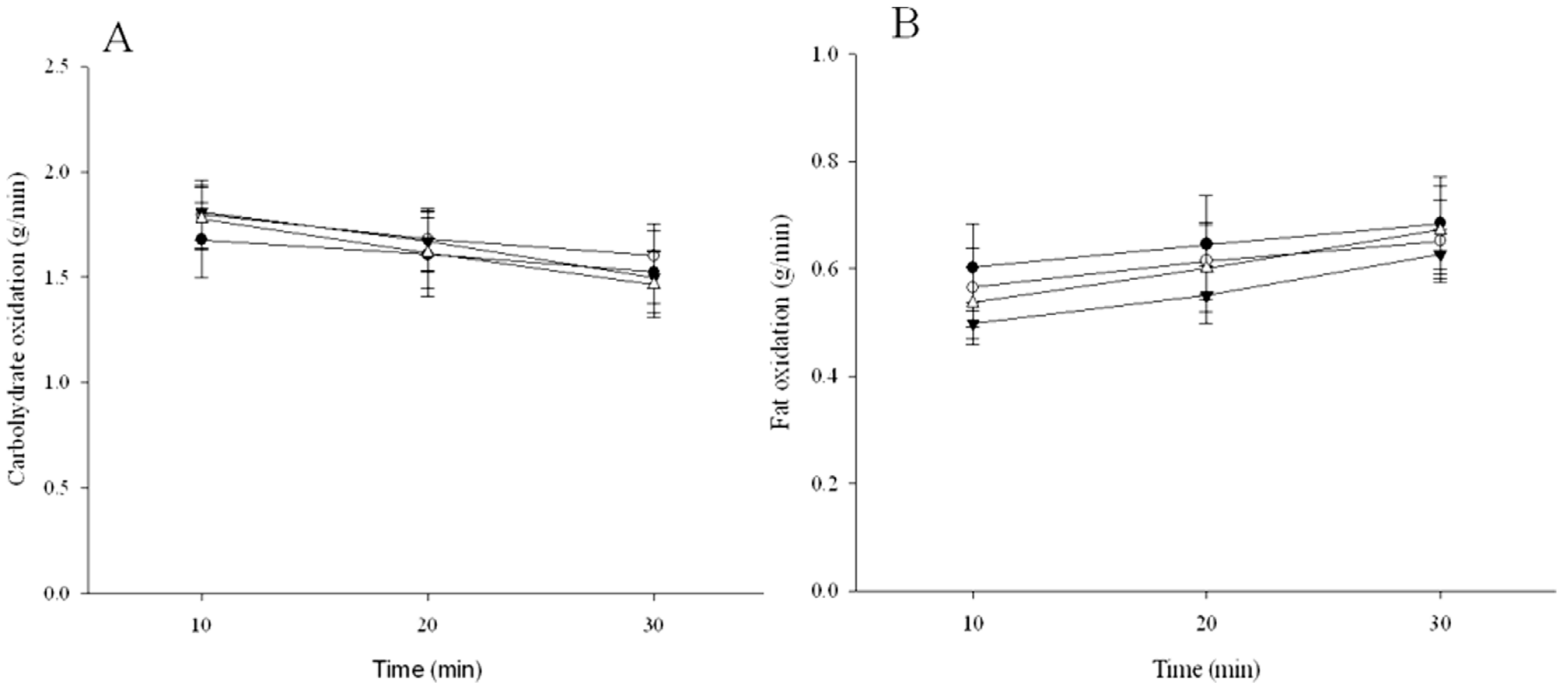
Carbohydrate oxidation (g/min) (A) and fat oxidation (g/min) (B) rates during 30 min steady state exercise (55% VO_2_ max) 1 hour following ingestion of caffeine, coffee, decaf or placebo beverages. Data represented seen as Closed circles – Caffeine Open circles – Caffeinated Coffee Closed triangles – Decaffeinated coffee Open triangles – Placebo. Means ± SE n = 8

Carbohydrate oxidation rates during SS significantly reduced in all treatments across time (P = 0.001). However there was no significant difference in carbohydrate oxidation between each of the treatments (Figure 1A P = 0.288). Similarly, fat oxidation rates significantly increased during SS in all treatments (P = 0.001). No significant difference in fat oxidation was observed between each of the treatments (Figure 1B P = 0.445). Accordingly the contribution of carbohydrate and fat to total energy expenditure during SS was not significantly different between any of the treatments (P = 0.463).

#### Plasma metabolite concentrations

**Figure 2:**
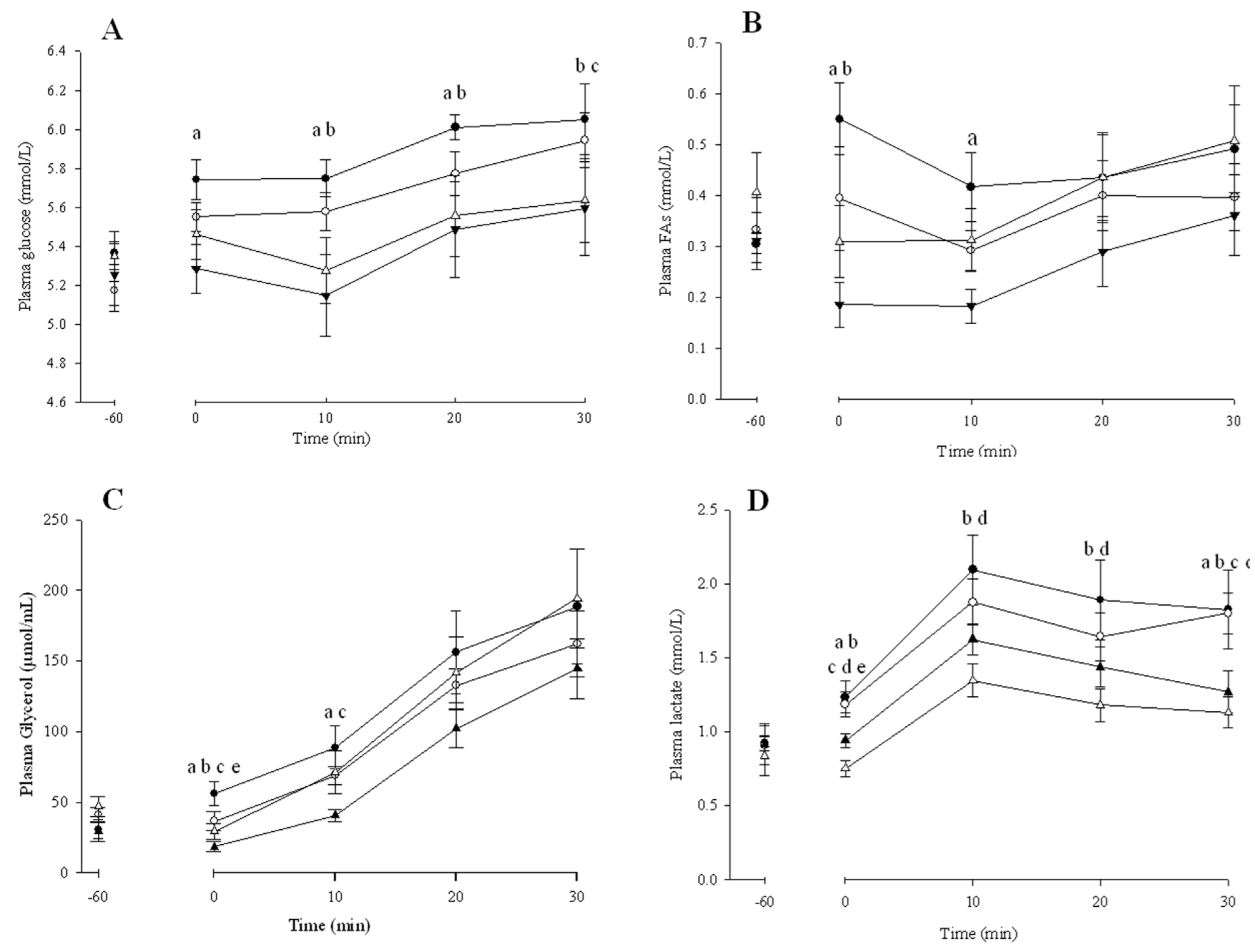
Plasma metabolite responses at rest (t = -60-0) and during 30 min steady state exercise (55% VO_2_ max) (t = 0–30) following ingestion of caffeine, coffee, decaf or placebo beverages. A Glucose. B Fatty acids (FA). C Glycerol. D Lactate. Data represented seen as Closed circles – Caffeine Open circles – Caffeinated Coffee Closed triangles – Decaffeinated coffee Open triangles – Placebo. a Sig. different between CAF and DECAF (p<0.05) b Sig. different between CAF and PLA (p<0.05) c Sig. different between COF and DECAF (p<0.05) d Sig. different between COF and PLA (p<0.05). Means ± SE n = 7.

Plasma metabolite responses at rest and exercise are displayed in Figure 2 A–D. Plasma glucose concentrations (Figure 2A) were significantly elevated at the end of rest compared to the beginning of rest following CAF and COF (p<0.05 for both), while no significant difference occurred following DECAF or PLA (P = 0.676 and 0.188 respectively). The elevation in glucose concentrations with CAF following the rest period was significantly higher compared to DECAF only (P<0.05). During exercise, plasma glucose increased over time following CAF and COF, however only COF reached statistical significance (P<0.05). DECAF and PLA glucose concentrations fell during the onset of exercise with a significant increase in both treatments later in exercise (T = 10–30 P<0.05 for both). As a result, CAF had significantly higher glucose concentrations within the first 20 minutes of exercise compared to DECAF and PLA (P<0.05 for both), while at end of exercise CAF and COF had significantly higher glucose concentrations compared to PLA only (P<0.05 for both). Plasma FAs concentration (Figure 2B) were significantly elevated at the end of rest compared to beginning of rest following CAF (P = 0.010), while DECAF and PLA had reduced FA concentration, with only DECAF reaching statistical significance (P = 0.007 and P = 0.072 respectively). Therefore, CAF had significantly higher FAs concentration compared to DECAF and PLA at end of rest period (P<0.05 for both). During the beginning of exercise, CAF continued to have significantly elevated FAs concentration compared to DECAF only (P = 0.037). FA concentration significantly increased during exercise (T = 10–30 P = 0.030) with no significant differences observed between treatments (P = 0.231).

Plasma glycerol concentrations (Figure 2C) did not significantly change at rest for CAF (P = 0.066), COF (P = 0.392) and DECAF (P = 0.104) but PLA significantly fell (P = 0.022). DECAF was significantly lower at end of rest compared to CAF, COF and PLA (P<0.05 for all), with no significant differences observed between any other beverage. During exercise there was a significant increase in glycerol concentrations for all treatments over time (P = 0.001), with significantly higher concentrations observed for CAF (P = 0.027) and COF (P = 0.003) at beginning of exercise compared to DECAF only. Plasma lactate concentrations (Figure 2D), were significantly increased following the consumption of CAF and COF only during the rest period compared to DECAF (P = 0.050 and P = 0.003 respectively) and PLA (P = 0.002 and P = 0.001 respectively). In addition DECAF had significantly elevated lactate compared to PLA at end of rest period (P = 0.012). All treatments lactate concentrations significantly increased at the onset of exercise (P = 0.004) with CAF and COF being significantly higher compared to PLA (P = 0.037 and P = 0.010 respectively). CAF and COF had sustained lactate concentrations at the end of exercise, with significantly higher concentrations compared to DECAF (P = 0.008 and P = 0.028 respectively) and PLA (P = 0.050 and P = 0.005 respectively).

#### Plasma caffeine concentrations

**Figure 3:**
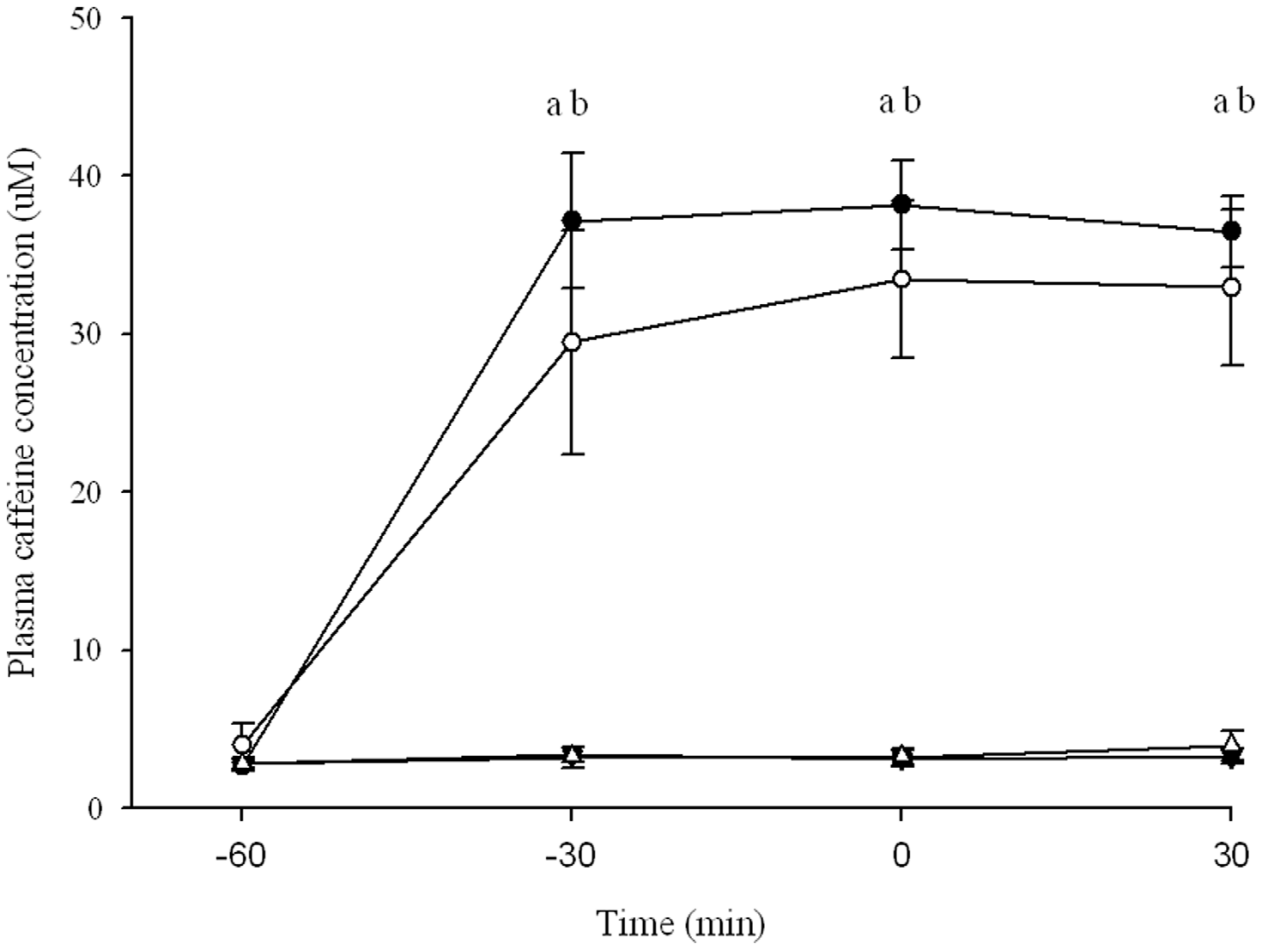
Plasma caffeine concentrations following ingestion of caffeine, coffee, decaf or placebo beverages. a CAF significantly different to DECAF and PLA (p<0.001) b COF significantly different to DECAF and PLA (p<0.05). Data represented seen as Closed circles – Caffeine Open circles – Caffeinated Coffee Closed triangles – Decaffeinated coffee Open triangles – Placebo Means ± SE n = 7.

The plasma caffeine concentrations following each beverage are displayed in Figure 3. At baseline plasma caffeine concentrations were very low for all treatments (<3 µM), with no significant differences observed (P = 0.478). Plasma caffeine significantly increased following CAF and COF when compared to DECAF (P = 0.000 and P = 0.009 respectively) and PLA (P = 0.000 and P = 0.010 respectively), with peak concentrations observed 60 min after intake (38.2±2.8 µM and 33.5±5.0 µM respectively). No significant difference was observed in the plasma caffeine concentrations between CAF or COF (P = 0.156) and DECAF or PLA (P = 0.558) throughout the trials.

### Time trial performance

**Figure 4:**
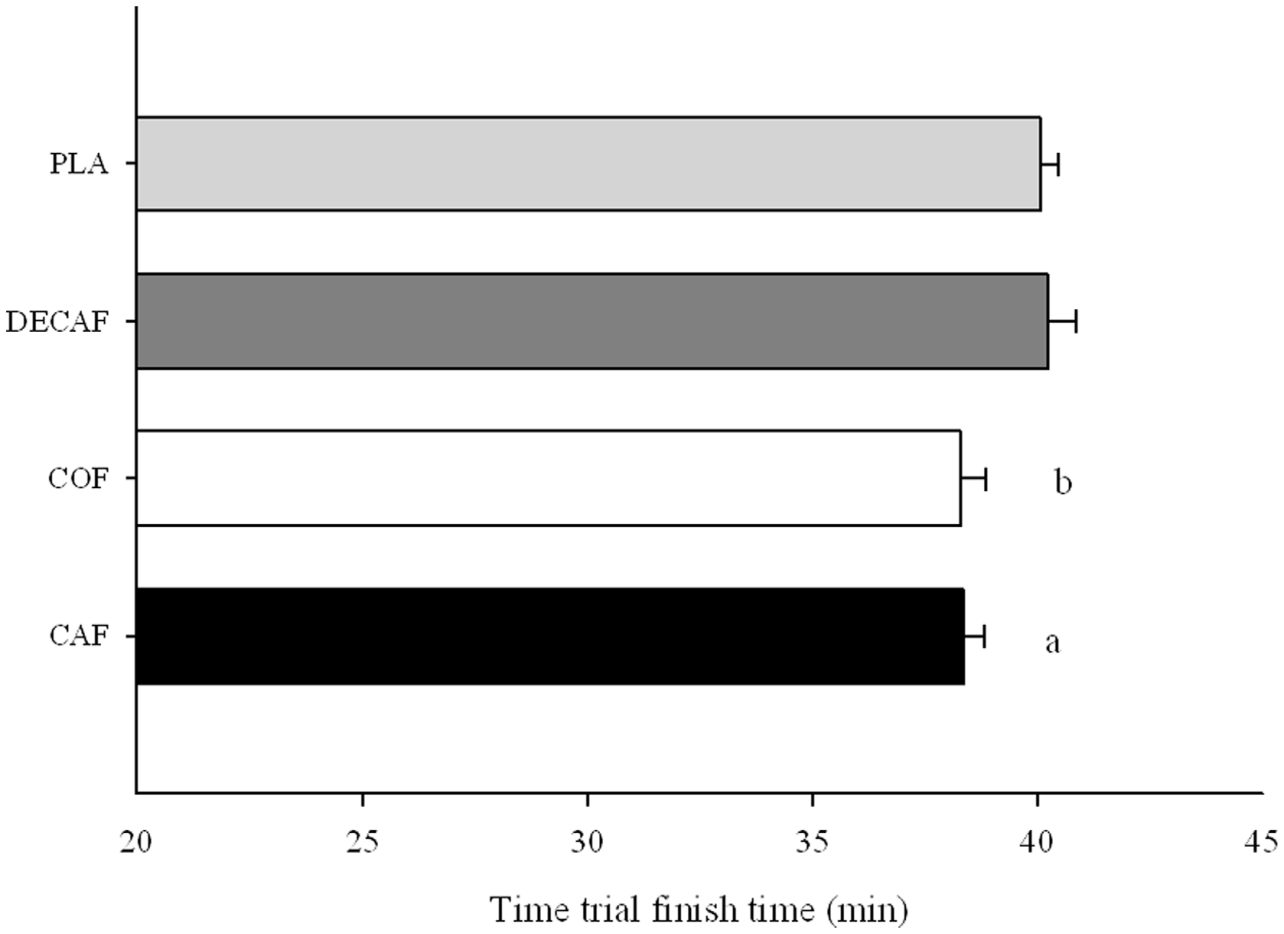
Time trial finishing time (min) for caffeine, coffee, decaf or placebo beverages a CAF significantly different to DECAF and PLA (p<0.05) b COF significantly different to DECAF and PLA (p<0.05). Data represented seen as Closed bar– Caffeine Open bar – Caffeinated Coffee Dark grey bar– Decaffeinated coffee Light grey bar– Placebo. Means ± SE n = 8.

CAF and COF significantly improved TT finishing times when compared to both DECAF (P<0.05 for both) and PLA (P = 0.007 and P = 0.010) (Figure 4). As a result mean power output during the TT was significantly greater for both CAF and COF compared to DECAF and PLA (294±6, 291±7, 276±7, 277±4 W, respectively P<0.05 for both). However no significant differences were seen in average heart rate during the TT between CAF, COF, DECAF and PLA (170±3, 167±4, 164±3, 165±4 BPM, respectively P = 0.516). CAF significantly improved TT performance by 4.9% (95% confidence interval (CI) = 2.3–6.8%) and 4.5% (95% confidence interval (CI) = 2.3–6.2%) compared to PLA and DECAF respectively (p<0.05 for both). Equally, COF significantly improved TT performance by 4.7% (95% confidence interval (CI) = 2.3–6.7%) and 4.3% (95% confidence interval (CI) = 2.5–7.1%) compared to PLA and DECAF respectively (p<0.05 for both) (Table 2). In addition there were no significant differences in TT finishing time between CAF and COF (P = 1.000) or PLA and DECAF (P = 1.000).

**Table 2:** Time trial performance data for each treatment.

Treatment	TT finish time (min)	Improvement compared to PLA % (95% confidence intervals)	P value	Improvement compared to DECAF % (95% confidence intervals)	P value
**CAF**	38.35±0.48^a^	4.9 (2.3−6.8)	0.007	4.5 (2.3−6.2)	0.012
**COF**	38.27±0.57^b^	4.7 (2.3−6.7)	0.010	4.3 (2.5−7.1)	0.012
**DECAF**	40.23±0.63	−0.4 (−4.0−3.1)	1.000	-	-
**PLA**	40.06±0.39	-	-	0.3(−0.3−3.9)	1.000

Means ± SE n  =  8 a significantly different to DECAF and PLA (p<0.05) b significantly different to DECAF and PLA (p<0.05) Abbreviations: CAF Caffeine, COF Coffee, DECAF Decaffeinated Coffee, PLA Placebo.

Following the completion of the TT, 3/8 participants were able to successfully guess the correct order of test beverages consumed prior to the trial. The correct guesses were more consistent for detecting CAF compared to the other drinks, with 6/8 of the participants guessing correctly. None of the participants reported any serious symptoms of GI distress at the end of any of the trials.

## Discussion

The present study examined the effects of acute intake of coffee (5 mg CAF/kg BW) and caffeine (5 mg CAF/kg BW) on time trial cycling performance, as well as substrate utilisation during SS exercise. Numerous studies to date have shown the efficacy of acute caffeine ingestion for improving prolonged endurance exercise performance [1]–[7]. The effects of caffeine on time trial endurance performance (>5 min) have recently been reviewed in a well conducted meta-analysis [7]. The authors concluded that of the 12 studies that investigated caffeine intake (1–6 mg CAF/kg BW), performance was improved by ∼3%. Fewer studies have investigated the ergogenic effects of coffee, with results being mixed thus far. In agreement with the literature, the current study found an improvement in performance following caffeine intake of 4.9% and 4.5% when compared to decaf coffee and placebo, respectively (Table 2). Interestingly, the current study also showed that coffee improved performance to the same extent as caffeine when compared to decaf coffee and placebo, 4.7% and 4.3% respectively. Thus, this is the first study to date to demonstrate that coffee consumed 1 h prior to exercise, at a high caffeine dose (5 mg CAF/kg BW), is equally as effective as caffeine at improving endurance exercise performance.

Our findings are in line with a number of studies that have shown improvements to performance following coffee intake [9], [19]–[21]. Costill et al [9] were the first to show that decaf coffee plus caffeine (330 mg), improved exercise time to exhaustion (80% VO_2_ max) compared with decaffeinated coffee (∼18%). More recently, Wiles et al [19] showed that coffee was able to improve 1500 m treadmill running performance when compared to decaffeinated coffee (∼3%). However, the current study results are in contrast to a number of other studies [22]–[24]. For example, the work conducted by Graham et al [22] showed that coffee (4.5 mg CAF/kg BW), regardless of the format of intake (regular coffee or decaffeinated coffee plus caffeine) did not result in an improvement to running time to exhaustion (75% VO_2_ max), where as caffeine (4.5 mg CAF/kg BW) significantly improved performance. Therefore it was concluded by Graham et al [22] that the performance effects of coffee may be inferior to caffeine. Despite this evidence, the current study clearly demonstrates that coffee is as effective as caffeine at improving endurance exercise performance.

The discrepancy in the performance effects of caffeine and coffee between the present study and Graham et al [22] might be explained by the type of performance test implemented. Time to exhaustion tests have been shown to be highly variable from day to day, with a coefficient of variation (CV) ∼27% in one study [18]. It is possible that this large variability may have contributed to the lack of performance effects found by Graham et al [22]. Whereas using a time trial performance measure, as used in the current study, has previously been shown to be highly reproducible (CV∼3%) and could detect smaller differences in performance [18]. Also the number of comparisons in the study by Graham et al [22] was greater than in the present study with a similar subject number, indicating that their statistical power was smaller. Perhaps for these reasons, the current study was able to detect similar changes in performance following caffeine and coffee intake (∼5%) (Table 2), whereas Graham et al [22] did not.

The composition and preparation of coffee in each of the studies [9], [19]–[24] may also explain the discrepancies in the ergogenic effects of coffee. Coffee is ∼2% caffeine, with the remainder composed of chlorogenic acids, ferulic acid, caffeic acid, nicotinic acid as well as other unidentifiable compounds [26]. It is evident that the source of coffee beans, roasting, storage and preparation (brewing and filtering) dramatically alters the caffeine and chlorogenic acid content of the coffee [26]. In accordance, recent evidence has shown that the chlorogenic acid content of commercially available espresso coffees range from 24–422 mg/serving [26]. In support, the current study observes a high chlorogenic acid content in both coffee and decaffeinated coffee samples (Table 1). Graham et al [22] speculated that chlorogenic acids found in coffee may have blunted the physiological effects of caffeine, preventing an improvement in exercise performance. However the authors did not report measurements of chlorogenic acids in coffee or in plasma to support this speculation. Despite the compounds present in coffee, the authors reported that the bioavailability of plasma caffeine and paraxanthines did not differ to caffeine [22], which is in line with the present study (Figure 4). Further, *in vitro* studies suggest that chlorogenic acids antagonize adenosine receptor binding of caffeine [27] and cause blunting to heart rate and blood pressure in rats [28]. Yet, *in vivo* there is no evidence to suggest that chlorogenic acids, especially at the low nanomolar concentration typically observed [32], impact on the mechanisms of action of caffeine that lead to the ergogenic effects. In support of this notion, and in agreement with the current study, regular coffee (1.1 mg/kg/BW) consumed prior to the ingestion of different doses of caffeine (3–7 mg/kg/BW) has been shown not to affect the ergogenic effects of caffeine [21].

The improvement in performance in the current study is unlikely to be explained by alterations to fat oxidation, as no difference during the SS exercise bout was observed (Figure 1B). This is in agreement with a number of investigations that do not support the thesis that caffeine improves exercise performance by augmenting fat metabolism [33], [34]. In addition these effects are apparent despite consistent increases in adrenaline though activation of the SNS [33], [34] and a subsequent elevation in FA appearance in the circulation following caffeine intake [33], [34]. It is evident that the improvement in performance is likely through caffeine's direct antagonism of adenosine receptors (*A_1_* and *A_2A_*) on the skeletal muscle membrane to improve excitation-contraction coupling [13] via a greater release of Ca^2+^ from the SR [16] and/or improved Na^+^/K^+^ ATPase pump activity [15]. In support of this notion, Mohr et al [12] observed that tetrapelegic patients, who have an impaired sympathoadrenal response [35], showed that caffeine improved exercise performance, while RER did not change during an electrical stimulated cycling test. Further, the authors also observed a significant increase in FA and glycerol at rest and during exercise following caffeine intake, despite a lack of an adrenaline response. This is due to the fact that adenosine has been shown to inhibit lipolysis [36] and enhance insulin stimulated glucose uptake in contracting skeletal muscle *in vitro* [37]. In support, the current studyobserved a significant increase in plasma glucose, FA and glycerol concentrations following caffeine (Figure 2 A, B, C). In addition, the consistently reported elevation in adrenaline concentrations [33] combined with adenosine receptor antagonism following caffeine intake during exercise may work synergistically to activate glycogenolysis in exercising and non-exercising tissues [34] as well as adipose tissue/skeletal muscle lipolysis [33]. This supports the fact that the current study (Figure 2 D) and others have shown that caffeine increase plasma lactate concentrations at rest and during exercise [33], [34]. Though to date there is little supporting evidence that caffeine stimulates exercising skeletal muscle glycogenolysis [33], [38], with early studies showing a paradoxical glycogen sparing effect with caffeine [2]. The elevated lactate concentrations are more likely due to a reduced clearance by the exercising muscle and a greater release by non exercising tissues [33]. Consequently, due to the healthy participants tested in the current study it is likely that the adenosine receptor antagonism by caffeine plays a crucial role in inducing the ergogenic effects of caffeine while regulating the metabolite response synergistically with the SNS.

Interestingly, despite coffee producing similar ergogenic effects as caffeine, the metabolite responses were not identical (Figure 2). The current study observed that the significant increase in plasma glucose, FA and glycerol with caffeine was paralleled with an attenuated response for coffee, and a significantly blunted response with decaf coffee when compared to placebo (Figure 2 A, B, C). This is likely due to the compounds in coffee [39] inducing subtle effects on antagonism of adenosine receptors (*A_1_* and *A_2A_*) in a variety of exercising and non exercising tissues. In accordance, Graham et al [22] previously showed that coffee resulted in a blunted adrenaline response when compared to caffeine at rest in humans, which was attributed to chlorogenic acids antagonizing adenosine receptor binding of caffeine [27]. In addition nicotinic acid, a fatty acid ester found in coffee known to inhibit lipolysis, has been shown to lower FA concentrations in patients suffering from hyperlipidemia [40]. Chlorogenic acids are also believed to improve glucose uptake at the skeletal muscle when compared to caffeine [41], also by altering the antagonism of adenosine receptors. More recently, caffeic acid has been found to stimulate skeletal muscle glucose transport, independent of insulin, when accompanied with an elevation in AMPK *in vitro* [42]. Despite the aforementioned evidence, it remains unclear why compounds in coffee appear to modulate the metabolite response but not the ergogenic effects of coffee in the current study.

The current study provided a large bolus of caffeine in the form of anhydrous caffeine or coffee one hour prior to exercise (5 mg/kg BW). The chlorogenic acid content of the coffee beverages was different, which is worth highlighting as a potential limitation of the current study. Previous studies have failed to make comparisons between coffee and decaf coffee and instead have used decaf plus anhydrous caffeine [9], [19]-[21], [23], [43]. In addition these studies did not examine the chlorogenic acid content of the test beverages. Thus, the novelty of the current study was that the performance effects were investigated between caffeine and coffee, independent of the combined effects of decaffeinated coffee plus caffeine. Adding a decaf plus caffeine trial would have been successful in controlling for chlorogenic acid content of the beverage. However, firstly, investigating the effect of chlorogenic acids on the metabolic and performance effects of caffeine was not the primary aim of the current study. Secondly, and more importantly, the low nanomolar concentration of chlorogenic acids *in vivo* [32] is unlikely to impact on the mechanisms of action of caffeine when compared to the physiological effects observed *in vitro* from supra physiological concentrations of chlorogenic acids [27], [28]. Yet, as differences in the metabolic effects of caffeine compared to coffee were observed in the current study, it may be important for future studies to control for chlorogenic acid content in coffee beverages or additionally increase the dose of chlorogenic acids to raise the bioavailability *in vivo*. In turn this will provide further insights into the metabolic differences between caffeine and coffee. In conclusion, the present study showed that caffeine and coffee (5 mg CAF/kg BW) were both able to improve exercise performance to the same extent, when compared to both decaffeinated coffee and placebo. Our data does not support the notion that chlorogenic acids found in coffee impair the ergogenic effects of caffeine. However, the compounds found in coffee may alter the metabolic effects, as the current study observed differences between caffeine and coffee at rest and during exercise. It is yet to be determined if lower doses of caffeine, when ingested as coffee, offer the same ergogenic effects. This would offer a more applicable and realistic nutritional strategy for athletes.

## References

[1] Graham TE, Spriet LL (1995) Metabolic, catecholamine, and exercise performance responses to various doses of caffeine. J Appl Physiol 78: 867–874. 7775331 10.1152/jappl.1995.78.3.867

[2] Spriet LL, MacLean DA, Dyck DJ, Hultman E, Cederblad G, et al (1992) Caffeine ingestion and muscle metabolism during prolonged exercise in humans. Am J Physiol 262: E891–898. 1616022 10.1152/ajpendo.1992.262.6.E891

[3] Jenkins NT, Trilk JL, Singhal A, O'Connor PJ, Cureton KJ (2008) Ergogenic effects of low doses of caffeine on cycling performance. Int J Sport Nutr Exerc Metab 18: 328–342. 18562777 10.1123/ijsnem.18.3.328

[4] Irwin C, Desbrow B, Ellis A, O'Keeffe B, Grant G, et al (2011) Caffeine withdrawal and high-intensity endurance cycling performance. J Sports Sci 29: 509–515. 21279864 10.1080/02640414.2010.541480

[5] Desbrow B, Biddulph C, Devlin B, Grant GD, Anoopkumar-Dukie S, et al (2012) The effects of different doses of caffeine on endurance cycling time trial performance. Journal of sports sciences 30: 115–120. 22142020 10.1080/02640414.2011.632431

[6] Doherty M, Smith PM (2004) Effects of caffeine ingestion on exercise testing: a meta-analysis. Int J Sport Nutr Exerc Metab 14: 626–646. 15657469 10.1123/ijsnem.14.6.626

[7] Ganio MS, Klau JF, Casa DJ, Armstrong LE, Maresh CM (2009) Effect of caffeine on sport-specific endurance performance: a systematic review. J Strength Cond Res 23: 315–324. 19077738 10.1519/JSC.0b013e31818b979a

[8] Cox GR, Desbrow B, Montgomery PG, Anderson ME, Bruce CR, et al (2002) Effect of different protocols of caffeine intake on metabolism and endurance performance. J Appl Physiol 93: 990–999. 12183495 10.1152/japplphysiol.00249.2002

[9] Costill DL, Dalsky GP, Fink WJ (1978) Effects of caffeine ingestion on metabolism and exercise performance. Med Sci Sports 10: 155–158. 723503

[10] Chesley A, Howlett RA, Heigenhauser GJ, Hultman E, Spriet LL (1998) Regulation of muscle glycogenolytic flux during intense aerobic exercise after caffeine ingestion. Am J Physiol 275: R596–603. 9688698 10.1152/ajpregu.1998.275.2.R596

[11] Graham TE, Spriet LL (1991) Performance and metabolic responses to a high caffeine dose during prolonged exercise. J Appl Physiol 71: 2292–2298. 1778925 10.1152/jappl.1991.71.6.2292

[12] Mohr T, Van Soeren M, Graham TE, Kjaer M (1998) Caffeine ingestion and metabolic responses of tetraplegic humans during electrical cycling. Journal of applied physiology 85: 979–985. 9729573 10.1152/jappl.1998.85.3.979

[13] Tarnopolsky MA (2008) Effect of caffeine on the neuromuscular system--potential as an ergogenic aid. Applied physiology, nutrition, and metabolism = Physiologie appliquee, nutrition et metabolisme 33: 1284–1289. 10.1139/H08-121 19088790

[14] Doherty M, Smith PM (2005) Effects of caffeine ingestion on rating of perceived exertion during and after exercise: a meta-analysis. Scandinavian journal of medicine & science in sports 15: 69–78. 15773860 10.1111/j.1600-0838.2005.00445.x

[15] Mohr M, Nielsen JJ, Bangsbo J (2011) Caffeine intake improves intense intermittent exercise performance and reduces muscle interstitial potassium accumulation. Journal of applied physiology 111: 1372–1379. 21836046 10.1152/japplphysiol.01028.2010

[16] Tallis J, James RS, Cox VM, Duncan MJ (2012) The effect of physiological concentrations of caffeine on the power output of maximally and submaximally stimulated mouse EDL (fast) and soleus (slow) muscle. Journal of applied physiology 112: 64–71. 21979804 10.1152/japplphysiol.00801.2011

[17] Tarnopolsky M, Cupido C (2000) Caffeine potentiates low frequency skeletal muscle force in habitual and nonhabitual caffeine consumers. Journal of applied physiology 89: 1719–1724. 11053318 10.1152/jappl.2000.89.5.1719

[18] Jeukendrup A, Saris WH, Brouns F, Kester AD (1996) A new validated endurance performance test. Med Sci Sports Exerc 28: 266–270. 8775164 10.1097/00005768-199602000-00017

[19] Wiles JD, Bird SR, Hopkins J, Riley M (1992) Effect of caffeinated coffee on running speed, respiratory factors, blood lactate and perceived exertion during 1500-m treadmill running. Br J Sports Med 26: 116–120. 1623356 10.1136/bjsm.26.2.116 PMC1478936

[20] Trice I, Haymes EM (1995) Effects of caffeine ingestion on exercise-induced changes during high-intensity, intermittent exercise. International journal of sport nutrition 5: 37–44. 7749424 10.1123/ijsn.5.1.37

[21] McLellan TM, Bell DG (2004) The impact of prior coffee consumption on the subsequent ergogenic effect of anhydrous caffeine. International journal of sport nutrition and exercise metabolism 14: 698–708. 15657474 10.1123/ijsnem.14.6.698

[22] Graham TE, Hibbert E, Sathasivam P (1998) Metabolic and exercise endurance effects of coffee and caffeine ingestion. J Appl Physiol 85: 883–889. 9729561 10.1152/jappl.1998.85.3.883

[23] Lamina S, Musa DI (2009) Ergogenic effect of varied doses of coffee-caffeine on maximal aerobic power of young African subjects. African health sciences 9: 270–274. 21503180 PMC3074398

[24] Butts N, D C (1985) Effect of caffeine ingestion on cardiorespiratory endurance in men and women. Res Q Exerc Sport 56: 301–305.

[25] Desbrow B, Leveritt M (2006) Awareness and use of caffeine by athletes competing at the 2005 Ironman Triathlon World Championships. International journal of sport nutrition and exercise metabolism 16: 545–558. 17240785 10.1123/ijsnem.16.5.545

[26] Crozier TW, Stalmach A, Lean ME, Crozier A (2012) Espresso coffees, caffeine and chlorogenic acid intake: potential health implications. Food Funct 3: 30–33. 22130653 10.1039/c1fo10240k

[27] de Paulis T, Schmidt DE, Bruchey AK, Kirby MT, McDonald MP, et al (2002) Dicinnamoylquinides in roasted coffee inhibit the human adenosine transporter. European journal of pharmacology 442: 215–223. 12065074 10.1016/s0014-2999(02)01540-6

[28] Tse SY (1992) Cholinomimetic compound distinct from caffeine contained in coffee. II: Muscarinic actions. Journal of pharmaceutical sciences 81: 449–452. 1403678 10.1002/jps.2600810512

[29] Currell K, Jeukendrup AE (2008) Validity, reliability and sensitivity of measures of sporting performance. Sports medicine 38: 297–316. 18348590 10.2165/00007256-200838040-00003

[30] Borg G (1982) Psychophysical bases of perceived exertion. Med Sci Sports 14: 377–381. 7154893

[31] Jeukendrup AE, Wallis GA (2005) Measurement of substrate oxidation during exercise by means of gas exchange measurements. Int J Sports Med 26 Suppl 1S28–37. 15702454 10.1055/s-2004-830512

[32] Stalmach A (2012) Bioavailability of Coffee Chlorogenic Acids, in Coffee: Emerging Health Effects and Disease Prevention (ed Y.-F. Chu). Wiley-Blackwell, Oxford, UK

[33] Graham TE, Helge JW, MacLean DA, Kiens B, Richter EA (2000) Caffeine ingestion does not alter carbohydrate or fat metabolism in human skeletal muscle during exercise. J Physiol 529 Pt 3: 837–847. 10.1111/j.1469-7793.2000.00837.x PMC2270224 11118510

[34] Raguso CA, Coggan AR, Sidossis LS, Gastaldelli A, Wolfe RR (1996) Effect of theophylline on substrate metabolism during exercise. Metabolism: clinical and experimental 45: 1153–1160. 8781304 10.1016/s0026-0495(96)90016-5

[35] Van Soeren M, Mohr T, Kjaer M, Graham TE (1996) Acute effects of caffeine ingestion at rest in humans with impaired epinephrine responses. Journal of applied physiology 80: 999–1005. 8964766 10.1152/jappl.1996.80.3.999

[36] Kather H, Bieger W, Michel G, Aktories K, Jakobs KH (1985) Human fat cell lipolysis is primarily regulated by inhibitory modulators acting through distinct mechanisms. The Journal of clinical investigation 76: 1559–1565. 2997284 10.1172/JCI112137 PMC424129

[37] Vergauwen L, Hespel P, Richter EA (1994) Adenosine receptors mediate synergistic stimulation of glucose uptake and transport by insulin and by contractions in rat skeletal muscle. J Clin Invest 93: 974–981. 8132783 10.1172/JCI117104 PMC294012

[38] Graham TE, Battram DS, Dela F, El-Sohemy A, Thong FS (2008) Does caffeine alter muscle carbohydrate and fat metabolism during exercise? Applied physiology, nutrition, and metabolism = Physiologie appliquee, nutrition et metabolisme 33: 1311–1318. 10.1139/H08-129 19088793

[39] Beaudoin MS, Graham TE (2011) Methylxanthines and human health: epidemiological and experimental evidence. Handbook of experimental pharmacology: 509–548. 10.1007/978-3-642-13443-2_21 20859811

[40] Wahlberg G, Walldius G (1992) Effects of nicotinic acid treatment on fatty acid composition of plasma lipids and adipose tissue in hyperlipidaemia. Scandinavian journal of clinical and laboratory investigation 52: 547–553. 1411265 10.3109/00365519209090132

[41] Ong KW, Hsu A, Tan BK (2012) Chlorogenic acid stimulates glucose transport in skeletal muscle via AMPK activation: a contributor to the beneficial effects of coffee on diabetes. PloS one 7: e32718. 22412912 10.1371/journal.pone.0032718 PMC3296733

[42] Tsuda S, Egawa T, Ma X, Oshima R, Kurogi E, et al (2012) Coffee polyphenol caffeic acid but not chlorogenic acid increases 5'AMP-activated protein kinase and insulin-independent glucose transport in rat skeletal muscle. The Journal of nutritional biochemistry 10.1016/j.jnutbio.2011.09.001 22227267

[43] Butts N, Crowell D (1985) Effect of caffeine ingestion on cardiorespiratory endurance in men and women. Res Q Exerc Sport 56: 301–305.

